# Improving Post-discharge Practice of Kangaroo Mother Care: Perspectives From Communities in East-Central Uganda

**DOI:** 10.3389/fped.2022.934944

**Published:** 2022-07-13

**Authors:** Doris Kwesiga, Phillip Wanduru, Eric Ssegujja, Justine Inhensiko, Peter Waiswa, Linda Franck

**Affiliations:** ^1^Department of Health Policy, Planning and Management, School of Public Health, Makerere University, Kampala, Uganda; ^2^Department of Women's and Children's Health, Uppsala University, Uppsala, Sweden; ^3^Centre of Excellence for Maternal Newborn and Child Health Research, Department of Health Policy, Planning and Management, School of Public Health, Makerere University, Kampala, Uganda; ^4^Department of Global Public Health, Karolinska Institutet, Stockholm, Sweden; ^5^Department of Family Health Care Nursing, University of California, San Francisco, San Francisco, CA, United States

**Keywords:** kangaroo mother care (KMC), pre-terms, Neonatology, low and middle-income countries (LMIC), Uganda (Sub-Saharan Africa)

## Abstract

**Introduction:**

Kangaroo mother care (KMC) is among the most cost-effective and easily accessible solutions for improving the survival and wellbeing of small newborns. In this study, we examined the barriers and facilitators to continuity of KMC at home following hospital discharge in rural Uganda.

**Methods:**

We conducted this study in five districts in east-central Uganda, within six hospitals and at the community level. We used a qualitative approach, with two phases of data collection. Phase 1 comprised in-depth interviews with mothers who practiced KMC with their babies and caretakers who supported them and key informant interviews with health workers, district health office staff, community health workers, and traditional birth attendants. We then conducted group discussions with mothers of small newborns and their caretakers. We held 65 interviews and five group discussions with 133 respondents in total and used a thematic approach to data analysis.

**Results:**

In hospital, mothers were sensitized and taught KMC. They were expected to continue practicing it at home with regular returns to the hospital post-discharge. However, mothers practiced KMC for a shorter time at home than in the hospital. Reasons included being overburdened with competing domestic chores that did not allow time for KMC and a lack of community follow-up support by health workers. There were increased psycho-social challenges for mothers, alongside some dangerous practices like placing plastic cans of hot water near the baby to provide warmth. Respondents suggested various ways to improve the KMC experience at home, including the development of a peer-to-peer intervention led by mothers who had successfully done KMC and community follow-up of mothers by qualified health workers and community health workers.

**Conclusion:**

Despite wide acceptance of KMC by health workers, challenges to effective implementation persist. Amid the global and national push to scale up KMC, potential difficulties to its adherence post-discharge in a rural, resource-limited setting remain. This study provides insights on KMC implementation and sustainability from the perspectives of key stakeholders, highlighting the need for a holistic approach to KMC that incorporates its adaptability to community settings and contexts.

## Introduction

The World Health Organization (WHO) recommends kangaroo mother care (KMC) as a high-impact but a low-cost intervention that improves the survival and development of small babies in resource-limited settings (1, 2). The components of KMC include early, continuous, and prolonged skin-to-skin contact between the mother or a caregiver and a baby; exclusive breastfeeding; early discharge; and continued follow-up of the baby at home (3). Recent systematic reviews have demonstrated that KMC, when implemented consistently, results in a significantly lower risk of death for small babies (4, 5). Other notable benefits of KMC included reduced risk of infections, reduced risk of re-admission, and improved weight gain (4, 5). For mothers and their families, there were benefits like positive emotions, less pain, increased breastmilk production, increased esteem and sense of control of their situation, and parental role identity. The safety and affordability of KMC have also been proven (6).

Despite the benefits of KMC in improving survival among small newborns, its uptake remains low (7). A survey conducted in Uganda in 2014 among 17 health facilities across the country indicated that only one facility had integrated KMC into its routine care (8). Another study at one of the biggest referral hospitals in Uganda found that continuous skin-to-skin contact in the first week of birth was 3 hours, which is below the recommended 20 hours [9].

Reasons for the low uptake of KMC are fairly well-documented. A systematic review on barriers and enablers of KMC found that in low- and middle-income countries, the biggest challenges mothers faced included lack of help with implementing KMC, perceptions of negative health worker attitudes, and lack of awareness of KMC (10). Physical discomfort was another problem noted, especially pain and fatigue. Mothers complained about backache from sleeping upright and having to do it for up to 24 hours at a time (6, 10). An observational study conducted in Uganda highlighted challenges of practicing KMC within health facilities, and these included a lack of space to implement KMC because of overcrowding, a lack of privacy, and a lack of infrastructure like seats for KMC; thus, some women had to sit on the floor (9). Another systematic review found that facilities had no designated KMC space, inadequate beds and seats for KMC, and other supplies like baby wraps (11).

Although there has been advancement in the understanding of barriers and enablers of KMC within facilities, there is limited research on the continuity of KMC post-discharge in resource-limited settings like Uganda. Early discharge is a pertinent component of KMC, but this is most useful if there is continuity of KMC at home. Therefore, in this study, we aimed to examine the barriers and facilitators to continuity of KMC at home following hospital discharge in rural Uganda.

## Materials and Methods

### Study Design

This was a multi-method qualitative study with a phenomenological approach. We conducted in-depth interviews (IDIs), key informant interviews (KIs), and group discussions. The study had both facility and community-based components. It took place over 1 year, with data collection done in phases: phase 1 included IDIs and KIs conducted in December 2016 and January 2017 and phase 2 focused on group discussions from May to July 2017.

In the group discussions, we used the principles of participatory learning and action (PLA) (12), where the study team engaged mothers and their support persons in a deductive process of identifying solutions to the problem of sub-optimal implementation of KMC at home. As described in Table 1, we used various problem-solving techniques including a “problem tree,” a “spider diagram,” and a “net to keep me safe.”

**Table 1:** Group discussion approaches.

**Problem tree**	**Spider diagram**	**A net to keep me safe**
The “Why” question is asked until the root cause of the problem is identified. *Example*: Mothers complain about difficulties while practicing KMC. Why? *Steps* 1. Draw a trunk representing the core issue and the roots and rootlets representing direct and indirect causes, and branches representing direct and indirect effects 2. Identify the causes at one or several levels and leave out the effects 3. Restrict the analysis to major causes and effects only 4. Don't consider causes and effects that strengthen each other through direct or indirect connections 5. Ask why between four and five times to understand the immediate or underlying causes of the problem 6. Identify the effect of the identified problem and include them as tree branches and leaves. Probe particularly for failure in uptake and adherence/consistent practice of KMC	The spider diagram was used to provide a non-stigmatizing way of identifying key problems that mothers experience during the practice of KMC. In the process, it will assess which of these problems is greater, why, and what opportunities exist for KMC practice (facilitators). Step 1: Draw the shape of a spider. In the middle of the spider's body is where the topic, i.e., problems and opportunities for practicing KMC will be written. Discuss these Step 2: After the mothers are done with the drawing, a discussion about the drawing will follow, for example asking the mothers: • Which of the problems are easier or harder to address? • What type of action could be taken to address the problem? • Who should take the action?	The objective of this tool was to help the mothers identify and understand the networks and stakeholders that can be relied on for support during the practice of Kangaroo Mother Care (KMC). They also reflected on how to improve KMC practice. Introduction: When we are faced with the challenges of having to practice KMC as mothers, there are some people, groups, or organizations that we will always run to for help who will listen to us or give us support. These are like nets that will catch us when we fall. In this exercise, you will show us who these people are in your family, in your community, and in the health facility. The mothers should also suggest ways in which the KMC experience can be made better, within hospitals and at the community level, and who should be involved in these processes. Help them devise strategies on how to approach people on the net. *We did not do this exercise using an actual net on the ground as planned, but rather used discussions only

### Study Setting

The study was carried out in Busoga region, a predominantly rural area situated in east-central Uganda. It has a population of about three million or 10% of Uganda's population residing in an area of ~7,100 square miles. The Busoga region has 10 administrative districts: Iganga, Mayuge, Bugiri, Kamuli, Kaliro, Namutumba, Namayingo, Luuka, Buyende, and Jinja.

**Figure 1:**
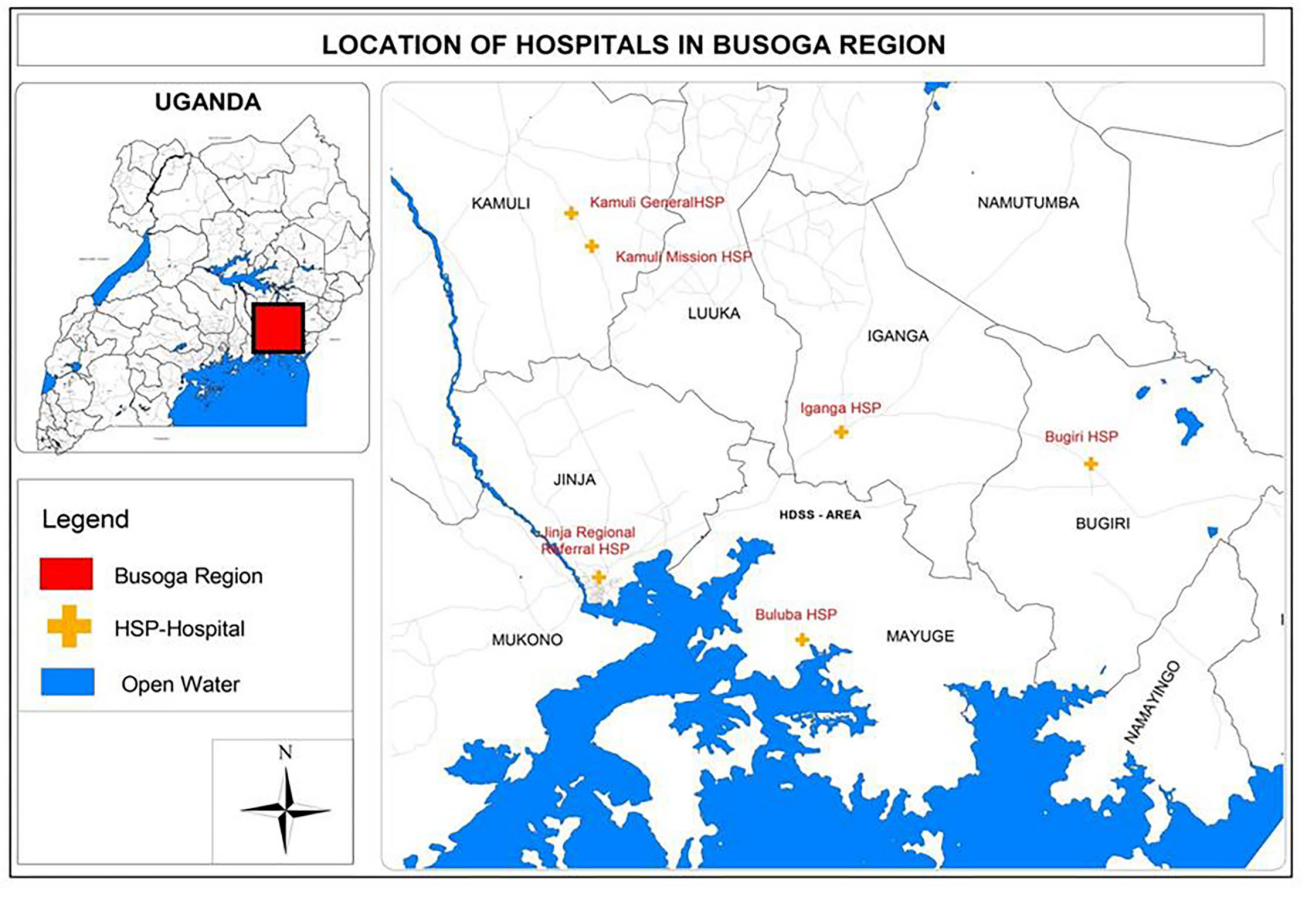
Hospitals and communities where study was conducted.

The facility-based component was conducted in six hospitals in eastern Uganda, which are a mix of four government- and two missionary-founded hospitals. These hospitals are spread out across five districts. Together, they had ~21,000 deliveries per year between 2016 and 2019. They all provide maternal and newborn services, including delivery and resuscitation of babies (Figure 1).

### Participant Selection

All participants, including mothers, support persons, community leaders, village health team (VHT) members, traditional birth attendants, district health officers (DHOs) in-charge of maternal and child health, and health workers were purposively selected. Mothers of pre-term or low-birth weight (LBW) babies were eligible to participate. Pre-term babies were defined as babies born before 37 completed weeks of gestation (13). Low-birth weight was defined as weight at birth less than 2,500 g (14). Mothers were selected from a pool of those who were practicing KMC in the hospitals at the time of the study and those who had practiced KMC in the past year in the hospitals (whether they completed it or not). Some were recruited in hospitals, where research assistants with the help of health workers recruited those who were practicing KMC at the time of the study from the wards. Others were identified when they came to the hospital for follow-up care for their babies. The last group of mothers who were at home were identified through the hospital record books and contacted using the telephone numbers they had registered.

Support persons were those who helped the mothers perform KMC after birth. These were family members, including siblings, husbands, and grandmothers. They were approached by the research assistants in person (those who were in the hospital with mothers at the time of the study) or by telephone, through the number given by the mothers. In some cases, mothers, often those who had twins or triplets, came for the group discussions with the support persons. Health workers were nurses in the six hospitals, specifically those in-charge of the maternity or newborn care unit (NCU) section in each hospital. Traditional birth attendants are those who offer informal “midwife” services in the communities, typically in their own homes. The research team was informed about these by different sources who knew about them—mothers who used them and VHTs. The VHT members are voluntary community health workers (CHWs) chosen by communities and trained by the government to offer support to health services in communities by providing certain basic healthcare, for instance, malaria treatment. The ones we interviewed were attached to the hospitals of study and were recommended by the health workers we interacted with. The DHOs supervise program implementation in the district, and we selected the assistant DHO in each district because they oversee maternal and child health.

Doctors were not among the study respondents. This is because, at the time of the study, none of them were involved in initiating, implementing, and/or supervising the implementation of KMC. Furthermore, it was difficult to get doctors to participate in interviews because none of them were assigned exclusively to the NCU; they only came in for brief periods of time to conduct clinical rounds before moving on to other parts of the hospital.

### Data Collection

In the communities, IDIs were conducted in the homes of the respondents. Some IDIs were held at the hospitals, with admitted mothers and those who came for follow-up care. The group discussions with mothers and support persons were held at or near the health facilities. The KIs with health workers were conducted at the health facilities. The VHTs were found at the health facilities, traditional birth attendants in their homes, and district health office staff in their places of work.

A group of seven research assistants, familiar with qualitative methodologies and with prior experience in maternal and child health research, conducted the IDIs and KIs. The group discussions were facilitated by three researchers also experienced in qualitative research methods, familiar with the study area, and with prior working knowledge of maternal and child health research and programming. The research assistants were trained on the study protocol before study commencement, and tools were discussed in both English and *Lusoga*, the most commonly spoken language in the study area. Interview guides informed by the literature and the study team's experience of the topic were developed by DK and reviewed by LF, PW, and ES for each of the different stakeholder groups. These were pre-tested, and minor revisions were made. Guides included questions on knowledge of care for pre-term/LBW babies; knowledge of KMC; the practice of KMC and the barriers and enablers therein; acceptability of KMC; and recommendations.

After the IDIs and KIs were completed, an initial analysis of the data collected was conducted to inform the group discussions. This resulted in slight modifications to the group discussion phase, followed by one group discussion in each district. The activities used to engage participants in group discussions (12) are elaborated in Table 1. All the interviews and group discussions were audio recorded, with field notes taken during the process after obtaining the consent of the participants. The interviews ranged from 40 min to 1 hour and 10 min in duration, while the group discussions lasted 2 hours. Upon completion of the interview, the audios were transcribed and translated into English, where this was not the study language. Finally, direct observations of KMC in the different settings (hospital and home) were conducted and documented in field notes by the researchers, with particular attention to the institutional, practical, and social support available to and used by mothers. Saturation was determined by the study team after having reached a point where no new insights were emerging and similar meanings were seen across the different data collection methods.

### Data Analysis

An interpretative paradigm was used in this study as well as thematic analysis (15). Inductive and deductive coding approaches were both used through an iterative process. Analysis of the IDIs and KIs was done by three people. While each coder worked independently, the team regularly held a series of meetings to discuss the codes and together agree on emerging themes; two of the coders used Atlas QI, a qualitative data management software to code the transcripts, while one conducted data analysis manually. For the group discussions, the first level of analysis was done during the discussion sessions with participants, followed by further analysis by the principal investigator (DK). Field and observation notes supplemented and integrated the analysis and the discussion section of this study.

All analyses aimed to discover commonly recurring themes and outliers. Triangulation was enhanced through the comparison of results between individual respondents, as well as between the different stakeholder groups and data collection methods, for areas of agreement and disagreement. Emerging themes were discussed among the study team members to identify underlying meanings. During the study team discussions, we analyzed topics for areas with greater consensus.

### Ethical Approval

This study received ethical approval from the Higher Degrees, Research and Ethics Committee at Makerere University, School of Public Health as well as Uganda National Council for Science and Technology. The study was conducted in accordance with the Declaration of Helsinki. All participants provided written informed consent before participation in the study. Efforts to protect the privacy and confidentiality of participants' identities and information were undertaken.

## Results

The results presented are from 65 respondents and five group discussions, whose details are shown in Table 2.

**Table 2:** Number of interviews and respondents.

**Category (A)**	**Number interviewed**
Mothers	14
Support persons/caregivers	10
Traditional birth attendants	6
Health workers	13
Assistant district health officers in charge of maternal and child health	5
Community leaders	7
Village health team members	10
Total	65
**District (B)**	**Number present in**
	**group discussion**
Jinja	8
Iganga	20
Kamuli	18
Mayuge	12
Bugiri	10
Total	68

### Knowledge About Kangaroo Mother Care Practice

Overall, the mothers understood KMC practice, reflected in their knowledge of its implementation. They reported that KMC refers to the wrapping of the baby skin-to-skin on the chest of the mother or any other caretaker in a way that positions the head to ensure breathing. The other critical aspects mentioned were monitoring the baby's temperature, breastfeeding or cup feeding when the baby cannot suckle, maintaining good hygiene, and returning to the health facility for follow-up appointments or when they noticed adverse signs. However, there were some gaps in knowledge among the mothers, for instance, in the number of hours KMC should be done, when exactly it should be terminated, and some of the other practicalities of KMC.

Three major themes that emerged as common and cutting across all stakeholder groups are presented below, with sub-themes discussed under each. These are (1) implementation of kangaroo mother care in health facilities—this is happening, but more support is needed; (2) continuity of kangaroo mother care at home post-discharge—this is predominantly difficult; and (3) recommendations to improve kangaroo mother care.

Although our study focuses on post-discharge, that part of KMC is a continuation of what occurs within the health facility. Therefore, we included a theme on the implementation of KMC in health facilities to present the basis of our study and explain the context, particularly for the reader who may not know what occurs in the Ugandan health facility context. We thus started with brief highlights of KMC in the health facilities and then focused on the community and the suggestions from participants.

### Theme 1: Implementation of Kangaroo Mother Care in Health Facilities

Our study indicates how KMC is initiated, the aspects of its implementation, and barriers to implementation in facilities. We found that KMC was always initiated by the health worker, after ascertaining the stability and health of the baby. The health worker then taught the mother about it and supported her to position the baby correctly on her chest. In all six hospitals, mothers and health workers revealed that KMC was done intermittently. Mothers revealed that they would do KMC for between 30 min and 2 hours continuously and then stop for a few hours before resuming. Most KMC was practiced in a special KMC room, although in one hospital, we observed mothers performing KMC in the special care unit (SCU). While mothers were predominantly the ones doing KMC, in the minority of cases, the sister to the mother of the pre-term helped her do KMC in the hospital, which was more common among those who had multiple births. Healthcare workers reported that occasionally, fathers would also actively get involved and perform KMC. One mother described her daily KMC routine:

*Yes, and I would do KMC and breast feed. For KMC, they would say 30 minutes and you give us our things. They would wrap the baby here [respondent demonstrates using her baby facing her how she used to do KMC] and they would tell me to go lie with it on the bed like this [demonstrates lying on her back] and it is here [in the chest]. From here [KMC], it would be taken back to their machine. (Mother, Iganga district)*

To support the implementation of KMC during admission, there was voluntary, informal peer-to-peer support, where mothers who had successfully started KMC supported and guided those who were new to it. This approach was encouraged by health workers as well.

*There is one instance a mother delivered a baby weighing 1.5 kg from a clinic and she brought it here because it wasn't feeding so I gave her an example from another mother, and they talked, and she liked the idea (Health worker, Mayuge district)*

Health workers in special care units (SCUs) thought positively about KMC as they had seen it produce favorable results. They therefore actively encouraged mothers to do it for small babies.

#### Barriers to Implementation of KMC in Health Facilities

Barriers to KMC implementation in the six healthcare facilities were classified into three main categories: human resource challenges, infrastructural inadequacies, and lack of support for mothers. The first two were mentioned by health workers, mothers, and support persons, while the third was mentioned by mothers and health workers.

Human resource challenges included understaffing, especially of NBUs and high staff turnover. Therefore, NBU staff could only dedicate a limited amount of time to teaching and monitoring the implementation of KMC for each mother and/or support person. This resulted in gaps in knowledge and practice of KMC in the NBU. In addition, hospitals have a policy of yearly rotations of nurses to other units. The incoming nurses were often not competent in KMC, and thus, there was a reduction in capacity to implement KMC after rotations. Most health workers reported that they had not been taught about KMC while still at school and therefore relied on externally organized refresher courses that targeted NBU staff.

*We had a workshop on care of preterm/LBW babies; this workshop took place I think around 2013 organized by Makerere MANeSCALE group and it was participatory, whereby we could use models then at the same time we came to the wards to practice it on these mothers because we had some mothers with preterm babies already in the ward. (Health worker, Kamuli district)*

Infrastructural inadequacies: Multiple challenges included small and crowded KMC rooms, few adjustable beds, and no special KMC chairs, which affected the comfort and the ability to do KMC for long hours. Mothers reported immense fatigue and pain from KMC as a result. Health workers explained that sometimes mothers had to be in the general maternity ward if the KMC room was full, and this was also observed in one hospital by the research team. Health workers explained that this was a potential source of infection for the babies. Poor hygiene and sanitation facilities also reduced the safety and effectiveness of KMC, and mothers protested about this.

Lack of support for mothers: Most mothers complained about the hospitals not providing food to patients, and this was corroborated by some health workers. They therefore had to look for their own food, and this was worse for mothers who were alone in the hospital because, as both mothers and health workers explained, they would have to stop KMC for a few hours to get food to eat, which affected their KMC.

### Theme 2: Continuity of Kangaroo Mother Care at Home Post-discharge

Discharge, according to some health workers, was on average done when the baby was stable and progressing and when they were confident that the mother had learned how to do KMC, usually two or more weeks after birth. However, in a few cases, mothers reportedly asked to be discharged early due to various challenges like not having a caretaker for their other children at home as well as the accumulating hospital bills. Prior to discharge, health workers explained that they encouraged mothers to continue KMC at home.

#### Implementing KMC at Home

Discussions with mothers revealed that on arrival at home, most of the women tried to practice KMC as advised. However, they reported that they were often unable to do it as much as they had done in the hospital. Mothers revealed that at home, KMC was done for fewer hours, although it was still skin to skin as taught. More so, they explained that they did KMC more consistently at night than during the daytime. Mothers also reported observing the required practices for hygiene maintenance and control of infections, including washing the baby's clothes frequently and limiting the number of people visiting. Predominantly, it was still done by the mother herself, except in one case where the father of the baby was helping his wife to do it at home. However, we also identified two mothers who said that they stopped practicing KMC as soon as they left the hospital.

Specific challenges to the continuity of KMC at home were clearly identified in the data. These are presented under three broad areas: lack of community follow-up after discharge, home/family-level challenges, and inadequate knowledge about KMC.

#### Lack of Community Follow-Up After Discharge

We learned that after discharge, there was no functional community-based follow-up system by health workers. According to the model of care offered when the study was done, health workers explained that mothers doing KMC were expected to return to the hospital to bring their babies for follow-ups, information confirmed by the mothers. Mothers and their babies were not followed up at home by health workers after discharge, predominantly due to a lack of resources to do so, as explained by health workers in the various districts. Additionally, health workers emphasized being overwhelmed with responsibilities in the hospital and not having time to do individual follow-ups. Mothers also reported that CHWs did not follow them up in the community either. Indeed, the CHWs we spoke to did not know much about KMC practice and did not highlight helping mothers at home with KMC as one of their roles.

Unfortunately, some babies got sick at home, before the date given for their return to the hospital, as mothers said, and as the research team observed in the field. More so, some mothers could not afford transport to return for care often, especially if they lived far from the hospital. This was mentioned by both the health workers and mothers. Indeed, health workers said that it sometimes led to the mother–baby pair dropping out of follow-up care at the health facility or coming less regularly. One health worker explained that by 6 months, very few mothers were bringing back babies for the scheduled checks, especially when the baby appeared to be doing well. Health workers explained that previously under some projects, the mothers were followed up in the community but once these projects ended, the process was unsustainable.

…*.when I was discharged, I was given appointment dates for going back in hospital to examine the baby, but they did not make follow-up at home to check on me…. (Mother, Kamuli district)*

#### Home/Family-Level Challenges

There were four main barriers to KMC practice at home. The first was the continuation of dangerous practices used to care for pre-term babies at home, despite KMC. Mothers and support persons revealed that they sometimes resorted to other interventions at home, beyond KMC. For instance, both in group discussions and IDIs with mothers and support persons, they explained that occasionally charcoal stoves were lit in the room in which the baby was, in order to provide warmth. Another common practice was putting hot water in plastic cans and placing these around the baby for warmth. Additionally, one mother reported giving herbs to her pre-term baby once she was home and abandoning KMC altogether. In a few instances, mothers and caretakers revealed that health workers had warned them against the use of charcoal stoves to produce warmth.

…*so, when I was discharged from hospital, I stopped doing it [KMC] and I even didn*'*t go for follow-ups though I was given appointment dates. According to me the baby was okay, so there was no need of going to hospital and struggling with that method of kangaroo mother care, I just used our local herbs, and my baby is healthy (Mother, Kamuli district)*

*You get water in a small jerrican, you put water here, there, and there [demonstrates on the ground how the water is put on all sides of the baby] and there is water on all the sides, and you put a charcoal stove nearby to make the baby warm with moderate fire (Mother, Iganga district)*

Gender roles and societal expectations toward mothers were other hindrances. Most women we interviewed in this study reported that they were expected to continue with their usual domestic chores once back at home, regardless of the fact that they had a pre-term baby to look after and needed to continue KMC. The majority of mothers described having to cook, clean, wash clothes, and look after animals and other children, as well as their husband/partner. Health workers corroborated the information about the heavy duties that the mothers had at home. Mothers sometimes asked to be discharged earlier, although they were still learning KMC because they had left their other children at home, and they needed to return to care for them. There was often only limited support for mothers doing KMC at home. Amid the many chores, most mothers explained that they did not have any social or in-kind support with this workload and health workers reiterated this. Being a predominantly rural setting, most mothers could not afford to hire somebody to help them at home with their chores. Those who had been supported by family members in the hospital often saw them depart on discharge, back to their own homes.

*There are some activities that out compete the skin-to-skin practice and the treatment that you could give to the baby. For example, taking goats to the field, washing clothes, cooking, fetching water, collecting firewood, maybe you have a business, and you want to work (Mother, Iganga district group discussion)*

Financial challenges were another key issue, which was particularly the case for mothers previously engaged in income-generating activities. Most mothers emphasized that KMC resulted in a loss of productivity and income on the part of the mother, who was often unable to engage in whichever economic activity she may have previously been doing. This was because KMC and care for a pre-term baby generally required a lot of time and commitment, and so the mother could hardly do anything else. There were a few outliers among participants, where men were actively involved in KMC and in supporting their wives/partners at home. While supportive of the mother's health and sustainment of KMC, this reduced the men's ability to work as regularly as they previously did, resulting in reduced income.

Mothers revealed challenges with the physical performance of KMC. The majority explained that it was painful to implement and was cumbersome for mothers who had undergone a cesarean section. It caused backache and chest pain and was tiresome. They explained that sitting upright or lying flat on the bed to do KMC increased pain and fatigue. These physical challenges led to mothers performing KMC for a shorter time, especially when there was inadequate support for KMC.

*Yes, there is real pain, too much backache, and you even feel it hard. The back really pains like a boil and even the chest pains and by time the baby is removed from the chest you are almost crying. (Iganga district group discussion)*.

As a result of the multiple difficulties mentioned, the mothers reported psychosocial challenges, including stress, depression, and discouragement. Health workers confirmed this as a major problem. The mother barely had time for her self-care, was often isolated with the baby, and barely socialized with other people.

#### Inadequate Knowledge About KMC

We found a gap in knowledge about KMC among the mothers, support persons, and community members. For example, some mothers and support persons were uncertain about the recommended number of hours KMC should be done, when it should be terminated, and how it should be done. In some cases, the mothers themselves also acknowledged their own inadequate KMC knowledge. Almost all mothers mentioned that their first interaction and exposure to KMC practice were at the health facility by the health workers after the birth of their index pre-term baby. Only two mothers indicated having heard about it before delivery of the index pre-term baby. One among these had a sister who previously gave birth to a pre-term baby, and the other was taught about KMC by a relative. Similarly, support persons had minimal information on KMC. Community members who were playing a role in maternal and newborn health, for instance, the traditional birth attendants and VHTs, were equally lacking in information about KMC.

### Theme 3: Recommendations to Improve Kangaroo Mother Care

As part of our participatory research approach to this study, we purposively explored the recommendations and preferences of community members for making KMC easier to implement and to ensure an increased uptake. Under this theme, we present three of the prominent improvements to address the uptake and sustainability of KMC as recommended by the participants.

#### Community Follow-Up

The follow-up of mothers in the community was noted as an enabler to better KMC uptake and acceptability by participants, including health workers, mothers, support persons, and VHTs. Among the suggestions on how the community follow-up could be done was the use of VHTs, including facilitating them with transport to do this job. As a health worker from Mayuge pointed out, the VHTs could conduct health education, while others agreed that they could check the progress of the pre-term baby. A VHT from Kamuli also agreed with VHTs following up the progress of mothers at home. The need for the VHTs to be trained on KMC so they can teach mothers within the community to do it as well as monitor their adherence was emphasized. However, the health workers supported VHT involvement only as part of the post-discharge care after the mother had learned about KMC in the hospital. They were not supportive of the idea of VHTs initiating KMC for babies born within the community, due to other intricate care needed for pre-term babies, especially within the first days of life.

Another suggestion for the follow-up from a caretaker was the option of health workers making phone calls to the mothers. The health workers were also supportive of the idea of health workers at lower level health facilities being involved in community follow-ups, provided that they received adequate training.

*Health workers should help and make home follow ups or making a call to check on the baby so that the person meant to practice it [KMC] must do it so when these babies keep on surviving then people will be motivated to practice the method (Caregiver, Kamuli district)*

#### Development of a Peer-to-Peer Intervention

Some of the mothers who had successfully done KMC and seen its positive effects on their babies eagerly recommended that they could assist and teach mothers who were new at KMC what to do. They volunteered and expressed their willingness to do so, including the provision of knowledge and practical demonstrations. Some health workers also felt that success stories of mothers who had successfully done KMC could be used as positive motivation and demonstration to others. One health worker envisioned having clinic days where mothers of pre-term or low-birth weight babies could come together and share their experiences, including KMC. Another health worker explained that at one point, they used to bring in mothers who had successfully done KMC to show others, but they feared this could spread infections to the babies and so stopped this.

*Now just like you have invited us, we can also go out and teach other women who are not yet in the system (Mother, Bugiri group discussion)*

*Even mothers that have practiced kangaroo should sensitize other people (Mother, Jinja district)*

#### Health Education and Training on KMC

Due to inadequate knowledge of KMC, mothers particularly recommended that pregnant mothers be taught during antenatal care about KMC, to avoid them having to hear of it for the first time when they had a pre-term birth. They also asked that husbands/partners/attendants be encouraged, for instance, through couple counseling to help them to do KMC. Health workers were supportive of teaching not only the mother but also those around her how to conduct KMC. Mothers and support persons also recommended raising awareness among communities, to sensitize them about KMC. They suggested different media, including radios, televisions, community meetings, posters in local languages for people to read, and integration of KMC into community outreaches on family planning. They said that this would in turn likely create more support among community members and reduce the burden of KMC being on the mother alone.

Several participants described the need to educate local council leaders for health and women and other political leaders about KMC and then have them as champions to support KMC programs and to encourage their communities to follow the advice of the health workers about KMC.

Training of more health workers on KMC was proposed by the health workers themselves. This included teaching all the relevant health workers KMC, including those not in SCUs, or providing refresher on-the-job training where needed. Many health workers were also supportive of having other cadres at health centers III and IV trained on KMC so that, where possible, they could provide it or initiate it and refer the baby in the KMC position.

## Discussion

This study set out to examine the barriers and facilitators to continuity of KMC at home following hospital discharge in rural Uganda. Results provide new insights into KMC practices at home in east-central Uganda, post-discharge, from the perspectives of a wide range of stakeholders across five districts. We have demonstrated that although key stakeholders including mothers, support persons, health workers, and other community leaders believe that KMC is important, practicing it is not always as simple or straightforward, especially after hospital discharge.

In all the facilities where the study was conducted, the mother–baby pair was discharged and expected to continue KMC at home. However, there was limited acknowledgment or preparation for how to maintain KMC within a setting very different from the closely monitored and regulated environment of the health facility. Many barriers that limit the consistent practice of KMC at home were described by study participants, for instance, lack of follow-up at home by health workers; the inability to quickly go back to the hospital in case of emergency; too many chores to do at home, often alone as per gender roles that diverted attention and time from doing KMC; financial challenges and reduced income; and inadequate knowledge; among others. These barriers resulted in less engagement with healthcare, KMC practiced less frequently, babies getting sick at home with parents unsure what to do next, high levels of psycho-social stress among mothers, and other adverse effects for mothers, babies, and the family.

Currently, there is a renewed global push to scale up KMC, including by WHO, which recently released results of a multi-country randomized controlled trial that reported the effectiveness of immediate KMC for small babies (2, 16). In Uganda, new guidelines for scale-up of KMC are under development. It is therefore a critical time to examine barriers and enablers of KMC practice following discharge from health facilities. It is important that the progress in improved infant survival and long-term health and developments that have been made through the implementation of KMC in hospitals are not lost after discharge. Indeed, many researchers have highlighted that community care must be linked to facility care in order to have successful KMC implementation (17–19).

Our study uniquely investigated and presented ideas for the improvement of KMC in the community from a range of key stakeholders. First, stakeholders recommended the provision of community follow-up by qualified health workers and CHWs. This recommendation is supported by previous research. Having a linkage and referral system between communities and providers via CHWs was identified as a potential enabler of KMC practice in Pakistan (1, 10, 20). In rural Malawi, a better follow-up of LBW babies after discharge was done through community follow-ups (21). An innovative CHW-led model of follow-up could be developed in Uganda, relying on the CHWs to do this monitoring, but with each CHW linked to a health facility or professional health workers whom they can contact for guidance. A blended workforce of health workers and CHWs may be needed to provide essential follow-ups for KMC support in the context of the human resource shortage in health that exists in Uganda and similar settings. However, this first requires that CHWs are taught about KMC practice, from initiation to danger signs and termination. Furthermore, funding is critical, for instance, to incentivize the CHWs in addition to thinking through how sustainability can be ensured.

Additionally, front-line, lower-level facility staff need to know about KMC so that they can either take part in the community outreach or more easily supervise it if CHWs are involved. The lower-level facilities are often more accessible to rural families, so if the baby needs a checkup or monitoring, they could be the first point of call before traveling to the hospital. This is also in line with our prior recommendations for newborn care to be strengthened at lower-level facilities (22).

Development of a peer-to-peer approach for KMC support would be complementary, with the mothers potentially more comfortable learning from other women with successful KMC stories, as recommended by the WHO (1). Peer-to-peer support in the community also potentially reduces the burden on the health workers and promotes knowledge transfer but may require some additional resources for sustainability. A systematic review identified enablers to KMC practice for caregivers, such as sharing positive testimonies of KMC by caregivers about its benefits; paternal involvement; peer support; trained nurses; and ability to do KMC at home (23).

Based on the findings from this study, we recommend advocacy and sensitization of communities about KMC and about care for small newborns with the goal of increased support for mothers and to enable families to continue KMC once back home, with fathers also encouraged to do KMC themselves. This could help reduce the stigma around pre-term and LBW babies and the expectations among many people that these babies will not survive. Families and communities should be educated about current survival and outcomes for pre-term and LBW newborns and the beneficial and harmful practices of caring for newborns, with emphasis on the necessity of abandoning dangerous practices like placing charcoal stoves in the same room as the baby. The use of charcoal stoves in the household has been linked to respiratory problems (24) and burn injuries (25). It appeared that KMC practice had not yet had enough impact on the eradication of harmful cultural practices. Communities must also be sensitized on the need to reduce the mother's workload once she is back home, as well as provision pf psycho-social support. They also need to be enlightened that KMC can be done by fathers as well as mothers.

Messages could be through traditional and social media, community meetings, posters in local languages, integrating KMC into community outreaches on family planning, among others. Local leaders should also be engaged to advocate for KMC programs. Similar strategies were successful in rural Malawi, with an integrated package of community-based sensitization interventions to reduce the stigma toward LBW babies (21). Further research will be needed to determine the effectiveness of these interventions in the Ugandan context, including determining the costs and acceptability of the interventions.

The strength of this study is that we provide experiences and perspectives from a wide variety of stakeholders/districts within a low-income setting. We also emphasize innovations suggested by the community members themselves through consensus, rather than by the researchers. We believe this could increase acceptability if these interventions are developed further. We were able to adapt the planned PLAs to meet the needs of the participants, especially since all mothers attended the group discussions with their babies, requiring adaptation of the activities into group discussions, with participants coming to a consensus on issues discussed.

## Conclusion

We used a novel approach to engage diverse stakeholder groups to explore KMC uptake and sustainability in eastern Uganda. While global advocacy continues for increased uptake and scale-up of KMC, we need to be cognizant of other factors that play a role in its success, beyond the medical process and the mother's knowledge. We have also reported the required supporting factors for successful KMC practice in the community and innovations suggested by community members themselves.

Kangaroo mother care is not only a medical issue; it has sociocultural, economic, family, and community components too. There is a need for a holistic approach, including consideration of the baby's life and health after discharge, within the particular context of the mothers and families' social, economic, and psychological welfare. However, due to the financial implications, innovative use of locally available resources is needed. Importantly, KMC, including community KMC, should be integrated into routine health systems and newborn care to ensure better survival and optimal development of newborns.

## Data Availability Statement

The raw data supporting the conclusions of this article will be made available by the authors, without undue reservation.

## Ethics Statement

The studies involving human participants were reviewed and approved by the Higher Degrees, Research and Ethics Committee at Makerere University School of Public Health and Uganda National Council for Science and Technology. The patients/participants provided their written informed consent to participate in this study.

## Author Contributions

DK, PWai, LF, and ES conceptualized the study design. DK, PWai, LF, ES, JI, and PWan contributed to the data collection, analysis, and drafting of the manuscript. All authors reviewed the manuscript and provided substantial input and approved the final manuscript.

## Funding

This study was supported by the University of California, San Francisco, Pre-term Birth Initiative-East Africa, an initiative funded by the Bill and Melinda Gates Foundation (Grant number OPP1107312). The funding body did not play any direct role in the design of the study, collection, analysis, and writing of the manuscript. The content is solely the responsibility of the authors.

## Conflict of Interest

The authors declare that the research was conducted in the absence of any commercial or financial relationships that could be construed as a potential conflict of interest.

## Publisher's Note

All claims expressed in this article are solely those of the authors and do not necessarily represent those of their affiliated organizations, or those of the publisher, the editors and the reviewers. Any product that may be evaluated in this article, or claim that may be made by its manufacturer, is not guaranteed or endorsed by the publisher.
